# Resilience, quality of life and symptoms of depression among elderlies receiving outpatient care*

**DOI:** 10.1590/1518-8345.3133.3212

**Published:** 2019-10-28

**Authors:** Gabriella Santos Lima, Isabela Maria Oliveira Souza, Luana Baldin Storti, Mônica Maria de Jesus Silva, Luciana Kusumota, Sueli Marques

**Affiliations:** 1 Universidade de São Paulo, Escola de Enfermagem de Ribeirão Preto, PAHO/WHO Collaborating Centre for Nursing Research Development, Ribeirão Preto, SP, Brazil.; 2 Scholarship holder to the Coordenação de Aperfeiçoamento de Pessoal de Nível Superior (CAPES), Brazil.

**Keywords:** Aged, Resilience Psychological, Quality of Life, Depression, Geriatric Nursing, Aging, Idoso, Resiliência Psicológica, Qualidade de Vida, Sintomas Depressivos, Enfermagem Geriátrica, Envelhecimento, Anciano, Resiliencia Psicológica, Calidad de Vida, Depresión, Enfermería Geriátrica, Envejecimiento

## Abstract

**Objective:**

to analyze the relation between resilience and demographic variables, quality of life and symptoms of depression in elderlies attended at a Geriatric Outpatient Clinic.

**Method:**

analytical cross-sectional study, conducted with 148 elderlies, with a questionnaire of sociodemographic and health characterization, the Resilience Scale, the World Health Organization Quality of Life Bref, the World Health Organization Quality of Life Old, and the Center for Epidemiologic Survey - Depression Scale. Descriptive statistics, Student’s t-test and Pearson correlation were used for data analysis.

**Results:**

there was a positive correlation between resilience and schooling (r = 0.208; p = 0.010), income (r = 0.194; p = 0.017), the World Health Organization Quality of Life Bref (r = 0.242; p = 0.003), and the World Health Organization Quality of Life Old (r = 0.522; p <0.001), and negative correlation regarding symptoms of depression (r = -0.270; p = 0.001).

**Conclusion:**

Resilience presented relation to schooling, income, quality of life and symptoms of depression in the elderly. These results are expected to help the multidisciplinary team plan actions aimed at developing resilience towards the promotion of health and good quality of life in old age.

## Abstract (pt)

**Objetivo:**

analisar a relação entre a resiliência e as variáveis sociodemográficas, a qualidade de vida e os sintomas depressivos dos idosos atendidos em um Ambulatório de Geriatria.

**Método:**

estudo transversal e analítico, realizado com 148 idosos, utilizando-se um questionário de caracterização sociodemográfica e de saúde, a Escala de Resiliência, o *World Health Organization Quality of Life Bref*, o *World Health Organization Quality of Life Old* e a *Center for Epidemiologic Survey - Depression*. Para a análise dos dados, utilizaram-se a estatística descritiva, o teste t-Student e a correlação de Pearson.

**Resultados:**

houve correlação positiva entre a resiliência e a escolaridade (r=0,208; p=0,010), a renda (r=0,194; p=0,017), o *World Health Organization Quality of Life Bref* (r=0,242; p=0,003) e o *World Health Organization Quality of Life Old* (r=0,522; p<0,001) e negativa com sintomas depressivos (r=-0,270; p=0,001).

**Conclusão:**

a resiliência apresentou relação com a escolaridade, a renda, a qualidade de vida e os sintomas depressivos dos idosos. Espera-se que esses resultados possam auxiliar a equipe multidisciplinar no planejamento de ações que visem à promoção da resiliência, com finalidade de promoção da saúde e boa qualidade de vida na velhice.

## Abstract (es)

**Objetivo:**

analizar la relación entre la resiliencia y variables sociodemográficas, calidad de vida y síntomas depresivos de los ancianos atendidos en un ambulatorio geriátrico.

**Método:**

estudio transversal y analítico en que participaron 148 ancianos y que utilizó un cuestionario de caracterización sociodemográfica y de salud, la Escala de Resiliencia, el *World Health Organization Quality of Life Bref*, el *World Health Organization Quality of Life Old* y la *Center for Epidemiologic Survey - Depression*. Para el análisis de datos, se utilizaron estadística descriptiva, la prueba t-Student y la correlación de Pearson.

**Resultados:**

hubo una correlación positiva entre resiliencia y escolaridad (r=0,208; p=0,010), ingresos (r=0,194; p=0,017), el *World Health Organization Quality of Life Bref* (r=0,242; p=0,003) y el *World Health Organization Quality of Life Old* (r=0,522; p<0,001), y una correlación negativa con síntomas depresivos (r=-0,270; p=0,001).

**Conclusión:**

la resiliencia se relacionó con el nivel de escolaridad, ingresos, calidad de vida y síntomas depresivos de los ancianos. Se espera que estos resultados puedan ayudar al equipo multidisciplinario a planificar acciones destinadas a impulsar la resiliencia, con el propósito de promover la salud y la buena calidad de vida en la vejez.

## Introduction

In the face of world population aging, the complexity of the biopsychosocial aspects of the human aging process becomes evident, which are often associated with physical, psychological and social overload in old age.

The presence of noncommunicable chronic diseases (NCDs) in the elderly can compromise their functional, cognitive, psychological, emotional, social and economic aspects, leading to disabilities, loss of autonomy and limitations that commonly correspond to a phase of life with negative connotation^(^ 1 ^)^. Given this, resilience, in its psychological concept, has been considered a fundamental factor to be developed throughout life^(^ 2 ^)^.

In old age, resilience is understood as the ability to cope, adapt and respond positively to changes that occur with advancing age. Psychologically resilient elderly do not surrender to adverse situations and, when facing them, demonstrate a capacity for positive adaptation, dealing with them and recovering their levels of subjective well-being^(^ 3 ^)^.

The elderly’s resilient characteristics and attitudes, associated with external resources, can provide good Quality of Life (QOL) in old age^(^ 1 ^)^. QOL in old age gathers typical dimensions of aging, such as environmental conditions (physical, ecological and man-made contexts), behavioral competence (performance against different life situations), perceived QOL (assessment of one’s own life, according to values ​​aggregated by personal and social expectations), and subjective well-being (satisfaction with one’s own global and specific life, between environmental and adaptive conditions)^(^ 4 ^)^.

The multifactorial nature of QOL has been related to demographic, clinical and behavioral factors^(^ 5 ^)^. Among the behavioral factors, resilient attitudes have been the target of research that seeks to understand their role and impact on QOL of various vulnerable groups, such as adults and/or elderly with or without NCDs; terminally ill people; people in situations of violence, abandonment and abuse, and people who have experienced natural disasters and civil war situations^(^ 6 ^-^ 7 ^)^. Nevertheless, there is still a lack of research focused on the evaluation of variables, resilience and QOL, relating them to the elderly living in the community and their respective risk and protection factors.

With regard to depression, it affected more than 300 million people worldwide in 2015, in different age groups, and predominantly among women^(^ 8 ^)^. In the same year, in Brazil, more than 11 million (5.8%) people aged 18 years or older were diagnosed with depression, with the largest proportion (11.1%) in the 60-64 age group^(^ 9 ^)^. The literature points out that, although depression often affects the elderly, it is less severe and prolonged and may last for years, with serious consequences for the emotional, psychological, functional and cognitive aspects of this population^(^ 10 ^)^.

The proportion and complexity of depression in the elderly population, which is often vulnerable by the process of human aging itself due to living with loss and illness, require physical and mental health care^(^ 10 ^)^. A review sought to assess the implications of resilience in the elderly population, and studies have suggested that resilient and coping profiles are relevant in the recovery from stressful events, also pointing out that low levels of depression and hopelessness are related to low levels of resilience^(^ 1 ^)^.

During old age, the elderly are susceptible to multiple risk factors for depression. It is believed that the elderly who have resilient reserves and characteristics, such as emotional control, optimism, maintenance of social relationships and autonomy, is able to achieve better outcomes in the management of situations that precede the symptoms of depression^(^ 11 ^)^. Other study results also showed an association between worse QOL and the presence of symptoms of depression^(^ 12 ^-^ 13 ^)^.

In addition to assessments and interventions directed to functional and cognitive changes and limitations imposed by the aging process, nursing care for the elderly is necessary when working with psychosocial aspects, which are susceptible to situations of vulnerability and changes in the state of health and disease^(^ 14 ^)^.

From a theoretical approach^(^ 15 ^)^, a reflective investigation of resilience in nursing praxis revealed that the resilience of people in chronic conditions presents itself as a knowledge gap to be explored by the science of care. In this this perspective, resilience is known as a promoter of human potentialities such as cognitive, behavioral, emotional, psychosocial and cultural skills, and it may be included in education and health promotion programs in relation to the age group most affected by NCDs.

Given the above, the results of the assessment of the resilient capacity of the elderly, related to quality of life and symptoms of depression, can strengthen the action strategies of the multidisciplinary team, especially nurses, regarding coping, adaptation and overcoming mechanisms in face of the of situations of disabilities and limitations that may arise with advancing age.

Therefore, the objective of this study was to analyze the relation between resilience and sociodemographic variables, QOL and symptoms of depression in the elderly attended at a Geriatric Outpatient Clinic of a Tertiary General Hospital, in the countryside of the State of São Paulo.

## Method

Analytical quantitative cross-sectional study performed at the Geriatric Outpatient Clinic of a Tertiary General Hospital in the countryside of the State of São Paulo. The population consisted of elderly people treated at the outpatient clinic, from May to October 2017, through semi-structured interviews, considering the pre-established inclusion and exclusion criteria.

Inclusion criteria: to be 60 years old or older; male or female; in attendance at the Geriatric Outpatient Clinic; to be able to communicate verbally and present preserved cognitive status assessed through the Mini-Mental State Exam (MMSE), considering the cutoff scores according to schooling levels^(^ 16 ^)^. The exclusion criterion: to be a resident in a Long-term Care Institution for the Elderly.

Consecutive non-probability sampling was used for sample selection^(^ 17 ^)^. Between May and October 2017, 614 elderlies were attended at the outpatient clinic, and 210 out of them were invited to participate in the study. Regarding these 210 elderlies, 14 were excluded (10 elderlies did not reach the minimum MMSE score according to their schooling, two had hearing impairment, one had mixed aphasia, and one was institutionalized), and 48 refused to participate in the study. Thus, the sample consisted of 148 elderlies.

The interviews were held on Tuesdays from 12:30 to 18:00, on the day and at the time of the Geriatrics Outpatient Service. To identify possible study participants, the schedule appointment listing was used to approach the elderly. At this time, the researcher and the research assistant introduced themselves, informed about the study and invited them to participate in the research. Then, they presented and discussed the Informed Consent Form and after each elderly agreed to participate, the signature was requested and a copy of the term was delivered to each one. The average duration of the interviews was 46.2 minutes.

For the interviews, a data collection instrument was used, containing: a) Questionnaire of Sociodemographic Characterization and Health Conditions, based on the sections of personal information, socioeconomic profile and health issues of the instrument prepared by the members of the Research Center of Geriatric and Gerontology Nursing, Ribeirão Preto School of Nursing, University of São Paulo (EERP/USP); b) World Health Organization Quality of Life Bref (WHOQOL-BREF), developed by the WHOQOL Group of the Division of Mental Health, aimed at evaluating the individual’s QOL, in the cultural context, value systems, personal goals, patterns and concerns. The instrument has 26 questions, two questions assess the overall QOL, and the remaining questions represent each of the 24 facets that make up the original instrument. The questions are grouped into four domains (physical, psychological, social relationships and environment) that assess the specific aspects of personal life. A Likert-type response scale was used, with scores from 1 to 5 for each question, which were transformed into a linear scale ranging from 0 to 100, where zero corresponds to the worst QOL and 100 to the best^(^ 18 ^)^. This instrument has been translated and validated in Brazil^(^ 18 ^)^. The validation of the Brazilian version showed adequate internal consistency by Cronbach’s alpha of 0.77 for the domains, and 0.91 for the questions^(^ 18 ^)^; c) World Health Organization Quality of Life Old (WHOQOL-OLD), which is a specific module for the evaluation of the elderly’s QOL, composed of 24 questions, with a Likert-type response scale, distributed in six facets (sensory abilities; autonomy; past, present and future activities; social participation; death and dying; intimacy). Each of the six facets has four items and it is possible to obtain values ​​between 4 and 20 points in each facet. The sum of the facet scores results in the overall QOL score in the elderly, and the higher the score, the better the QOL^(^ 19 ^)^. The Brazilian version was translated and validated^(^ 19 ^)^, and presented internal consistency represented by Cronbach’s alpha coefficient of 0.71 for facets, and 0.88 for overall QOL^(^ 19 ^)^; d) Center Epidemiologic Survey - Depression Scale (CES-D), originally designed for a North American epidemiological study^(^ 20 ^)^, with the purpose of detecting symptoms of depression in adult populations through self-reported depression-related symptoms. It investigates the frequency of symptoms of depression that have occurred in the week prior to the interview. The internal, construct and criterion validities of CES-D was performed in the elderly Brazilian population^(^ 21 ^)^. This scale has three domains, that is, negative affects, problems initiating behavior, and positive affects, distributed in 20 items, with a Likert-type response scale, with 0 (never or rarely); 1 (few times); 2 (most of the times), and 3 (always). The total score ranges from 0 to 60 points. The internal consistency by Cronbach’s alpha coefficient was 0.80 for negative affects, 0.68 for problems initiating behavior, and 0.63 for positive affects^(^ 21 ^)^. A cutoff score greater than 11 points differentiates cases of symptoms of depression from non-cases of symptoms of depression^(^ 21 ^)^, also adopted in this study; e) Resilience Scale (RS)^(^ 22 ^)^, which was adapted and validated for the Brazilian context^(^ 23 ^)^ to identify the degree of individual resilience, considering the positive personality characteristics that improve individual adaptation. The RS consists of three domains: resolution of actions and values ​​(which give meaning to life, such as friendship, personal fulfillment, satisfaction, and meaning of life); independence and determination; self-confidence and adaptability. The 25 items of the original scale were maintained using Likert-type responses ranging from 1 (strongly disagree) to 7 (strongly agree), resulting in scores ranging from 25 to 175 points, with high values indicating high resilience^(^ 23 ^)^, internal consistency was measured by Cronbach’s alpha coefficient of 0.80; intra-observer reliability for continuous variables was 0.74, and Kappa weighted for ordinal variables from mild to moderate^(^ 23 ^)^.

The data resulting from this study were tabulated using a Microsoft Office Excel^®^ spreadsheet, 2016 version, containing a codebook and two spreadsheets that were used for double-input validation (typing). After typing and validation, data were exported to the Statistical Analysis System^®^ software, version 9.4, for statistical analysis.

Data analysis was performed by means of descriptive statistics, Student’s t-test and Pearson’s correlation coefficient. To interpret the direction and strength of Pearson’s correlation coefficient (r), the following criteria were used (24): -1.0 (negative and perfect); -0.8 (negative and strong), -0.5 (negative and moderate); -0.2 (negative and weak); 0.0 (no association); 1.0 (positive and perfect); 0.8 (positive and strong); 0.5 (positive and moderate) and 0.2 (positive and weak). The significance level adopted in this study was 5%.

The project was submitted for consideration by the Research Ethics Committee of EERP/USP, obtaining approval, protocol CAAE No. 66567617.7.0000.5393. It was also submitted to the Research Ethics Committee of the General Hospital of Ribeirão Preto Medical School, University of São Paulo, a co-participating institution, with approval opinion, CAAE No. 66567617.7.3001.5440; both approved in May 2017.

## Results

A total of 148 elderlies participated in this study, and the majority, that is, 114, (77.0%) reported retirement as source of income. The average monthly income was 1,436.73 Brazilian *reais* (SD=1,142.27). Regarding health aspects, the mean of medical diagnosis per elderly was 7.4 (SD=7.4), and the most prevalent was systemic arterial hypertension (83.1%).

Table 1 shows the characterization of the elderly, according to age group, sex, marital status, schooling, and family arrangement.

**Table 1:** Distribution of the elderly, according to the variables age, sex, marital status, schooling, and family arrangement, attended at the Geriatric Outpatient Clinic, Ribeirão Preto, SP, Brazil, 2017

Variables	n	%	Mean (SD)*	Median	Amplitude (min-max)
Age group (years)			77.7 (6.8)	77.5	64- 92
60-79	90	60.8			
80+	58	39.2			
Sex					
Female	119	80.4			
Male	29	19.6			
Marital Status					
Married, living with the partner	53	35.8			
Widowed	78	52.7			
Divorced/Legally Separated/Separated	9	6.1			
Single	8	5.4			
Schooling (years)			3.9 (3.9)	3.0	0 – 20
Illiterate	28	18.9			
1 to 4	88	59.5			
5 to 8	14	9.5			
9+	18	12.1			
Family arrangement			2.3 (1.2)	2	0 – 6
Alone	30	20.2			
Only spouse	37	25.0			
Spouse and children	8	5.4			
Spouse, children, son-in-law or daughter-in-law	2	1.3			
Only the children	31	21.0			
Three-generation households	18	12.2			
Only the grandchildren	6	4.1			
Other^†^	16	10.8			
Total	148	100.0			

* SD = standard deviation;

† Other (elderly lives with: spouse and grandchild; brother and nephew; only brother; brother and parents; brother, sister-in-law and nephew; children; son-in-law or daughter-in-law).

In the QOL assessment, for WHOQOL-BREF, the overall QOL presented mean of 56.7 (SD=20.3), with the highest mean score for the “Social Relationship” domain, with 66.2 (SD=14.3), and the smallest for the “Physical” domain, with 50.7 (SD=15.4). In WHOQOL-OLD, the mean of overall QOL was 63.5 (SD=12.7). The “Death and Dying” facet had the highest mean, with 70.9 (SD=24.5), and the “Sensory Abilities” facet, the lowest mean, with 56.4 (SD=22.8).

Regarding symptoms of depression, verified by applying the CES-D scale, the elderly had a mean score of 16.5 (SD=10.7), and the majority, 95 (64.2%), had a score >11 points, indicating the presence of symptoms of depression.

The degree of resilience, assessed by the overall RS score, presented mean of 130.6 points (SD=18.0). It is noteworthy that the possible amplitude of the overall RS score is between 25 and 175 points, and the amplitude observed in this sample was between 85 and 175 points.

Table 2 shows the analysis of Pearson’s correlation coefficient (r) between the overall RS score and the sociodemographic variables (age, schooling, and income), the overall scores of WHOQOL-BREF and WHOQOL-OLD, and the CES-D score.

**Table 2:** Pearson’s correlation coefficient between the overall Resilience Scale score and sociodemographic variables, the overall scores of WHOQOL-BREF and WHOQOL-OLD, and the CES-D score of the elderly (n = 148) attended at the Geriatric Outpatient Clinic, Ribeirão Preto, SP, Brazil, 2017

Variables	Resilience Scale (Overall Score)	
	r	p-value*
Age	- 0.022	0.790
Schooling (years)	0.208	0.010
Income	0.194	0.017
Number of Diagnoses	0.077	0.349
WHOQOL-BREF	0.242	0.003
WHOQOL-OLD	0.522	<0.001
CES-D	- 0.270	0.001

* significant value p<0.05

It is observed that there was a positive and weak correlation between the overall RS score and schooling, income and WHOQOL-BREF. The correlation between the overall RS score and the overall WHOQOL-OLD score was positive and moderate. The correlation between the overall RS score and the CES-D score was negative and weak. The correlations between the mentioned variables were statistically significant, except for age and number of diagnoses.

Table 3 shows the comparison between the means of overall scores of RS, WHOQOL-BREF and WHOQOL-OLD for the sociodemographic variables and the presence or absence of symptoms of depression in the elderly studied.

**Table 3:** Distribution of the means and standard deviations of the scores of resilience and quality of life of the elderly (n = 148) attended at the Geriatric Outpatient Clinic, according to sociodemographic variables and symptoms of depression, Ribeirão Preto, SP, Brazil, 2017

Variables		Resilience Scale		WHOQOL-BREF		WHOQOL-OLD	
	n	Mean (SD*)	p-value^†^	Mean (SD*)	p-value^†^	Mean (SD*)	p-value^†^
Sex							
Female	119	130.3 (18.9)	0.720	55.2 (21.1)	0.034	63.3 (12.7)	0.675
Male	29	131.6 (14.4)		62.9 (15.8)		64.4 (13.1)	
							
Age group (years)							
60 to 79	90	130.1 (18.9)	0.685	55.5 (20.2)	0.373	62.7 (12.8)	0.307
80+	58	131.3 (16.8)		58.6 (20.5)		64.8 (12.5)	
							
Marital Status							
Without partner	95	132.4 (17.9)	0.092	58.5 (19.0)	0.172	64.5 (12.7)	0.200
With partner	53	127.2 (18.0)		53.5 (22.3)		61.7 (12.6)	
							
Schooling							
Yes	120	130.8 (18.4)	0.720	56.3 (19.7)	0.658	63.5 (13.1)	0.991
No	28	129.5 (16.5)		58.4 (23.3)		63.5 (11.0)	
							
Family arrangement							
Living Alone	30	138.3 (17.7)	0.010	62.9 (18.7)	0.054	67.8 (12.1)	0.037
Living with someone	118	128.6 (17.7)		55.1 (20.5)		62.4 (12.7)	
							
Income^‡^							
< 1 minimum wage	79	127.5 (18.4)	0.029	53.0 (21.1)	0.015	60.5 (12.2)	0.002
> 1 minimum wage	69	134.0 (17.1)		61.0 (18.7)		67.0 (12.5)	
							
Symptoms of Depression							
Yes	95	128.2 (18.2)	0.035	49.3 (19.8)	<0.001	59.4 (11.4)	<0.001
No	53	134.7 (17.2)		70.0 (13.5)		71.0 (11.6)	

* SD = standard deviation;

† p-value for Student’s t-test, significant value p<0.05;

‡ Minimum wage in force in Brazil in 2017 (R$ 937.00)

With regard to the RS, the elderly with higher mean scores had income higher than the minimum wage, without symptoms of depression, and lived alone. Regarding WHOQOL-BREF, the elderly who obtained higher means in the scale score were male, with income higher than the minimum wage, and without symptoms of depression. In relation to WHOQOL-OLD, the elderly who presented higher means lived alone, with income higher than the minimum wage, and without symptoms of depression.

## Discussion

During old age, resilience has been expressed as a protective factor for the elderly, possibly being a potential resource for changing habits and behaviors that offer a greater sense of well-being and QOL, besides maintaining physical and mental health^(^ 3 ^)^.

In this study, the sociodemographic characteristics of the elderly are similar to those found in other studies with Brazilian elderly receiving outpatient care. The mean for age was of 77.7 years^(^ 25 ^)^, there was predominance of females^(^ 26 ^)^, low schooling^(^ 27 ^)^, and widowhood^(^ 28 ^)^.

It is worth mentioning that 39.2% of the participants in this study were 80 years or older. This can be explained by the fact that increased longevity may be reflected in different behavioral, sociocultural, spiritual, belief and health patterns. A theoretical study, which addresses a broad discussion of resilience at an advanced age (85 years or older), found that older people live in a frequently vulnerable context, with variability of physical and mental conditions. However, having more years of life accompanied by potential gains, greater wisdom, experience, opportunities, a family and social support system, among others, can contribute to the development of coping mechanisms and adaptation to challenges and adversities^(^ 29 ^)^.

In this respect, the aspects that comprise resilient behavior and age groups, according to the literature findings^(^ 30 ^-^ 31 ^)^, are still inconclusive, as suggested by the results of this study, in which age was not associated with resilience.

This fact is corroborated by the dynamic perspective of resilience, which refers to resilient behavior that does not necessarily occur in a linear and progressive manner, and it is not a characteristic absolutely related to the life experience that is acquired with advancing age^(^ 32 ^)^. Regardless of a specific age group, resilient characteristics have been mediated mainly by the presence of social and family support, a favorable income condition, and good health.

Regarding schooling, there was a positive, weak and statistically significant correlation with the overall RS score. Schooling may be a factor that influences the way in which old age is experienced, as it goes beyond cognitive impairment and may have implications for the functional, psychological and socioeconomic aspects of the elderly. In this sense, resilience was explored by national studies in different contexts with trauma and illness situations^(^ 33 ^-^ 34 ^)^, and schooling also showed a significant correlation with resilience.

The presence of low schooling among the elderly reflects the national reality. The higher the age, the lower the schooling level^(^ 27 ^)^. Specifically, among the elderly, low education predisposes to unfavorable economic conditions, since they were in the informal market throughout their lives, with no planning and investment possibilities to guarantee retirement^(^ 35 ^)^.

The main source of income for the elderly in this study was retirement, with the average monthly income slightly exceeding a minimum wage in force at the time of data collection. Socioeconomic conditions, among other factors, determine housing, food, education and health options that are directly related to well-being and QOL. Moreover, a healthy lifestyle is common among older people with higher income, as it is often related to better lifestyle and ease of access to health services^(^ 36 ^)^.

Even in households with multigenerational arrangements, a significant portion of Brazilian families have the retirement of the elderly as their main source of income^(^ 37 ^)^. This fact may partially or totally compromise the elderly’s income, since their budget is burdened with expenses related to health care^(^ 37 ^)^. It should be emphasized that household arrangements that provide the interaction of the elderly with other family members and the social support network are factors that can contribute to the promotion of resilient characteristics^(^ 30 ^)^.

It is noteworthy that most of the elderly in this study lived with their spouses or children, but 20.2% of them lived alone. These elderly had higher means in RS, as well as in WHOQOL-BREF and WHOQOL-OLD. Different results were found in other studies^(^ 38 ^-^ 39 ^)^ conducted with the elderly, and those who had partners and who lived with more people had higher levels of resilience

The emphasis is thus on studies that consider relevant the social support offered by family members living in the same household as the elderly. It is extremely important for the elderly with high impairment of functionality and cognition, who need help of spouses, children and grandchildren^(^ 38 ^,^ 40 ^)^. However, it is necessary to consider the quality of family relationships and contexts that do not affect the quality of life during the old age, besides family members that respect the autonomy and independence of the elderly^(^ 40 ^)^.

According to the literature, there is an increase in the number of elderly people living alone. This can be explained by better health conditions, financial independence and autonomy for activities of daily living, as well as other factors such as reduced number of children per couple, widowhood and divorce^(^ 41 ^)^. Regarding these profiles, the resilient characteristics, which suggest greater ability to adapt to the limitations of aging, may be more present, so that the elderly, even living alone, feel strengthened, with their particularities, privacy and autonomy preserved in this phase of life^(^ 42 ^)^.

Another important factor, which occurs mainly in old age, is the frequent role of caregiver exercised by the spouse when the partner is affected by disabling disease sequelae, requiring the partner to help with hygiene, food and health care, which contributes to a scenario of physical and psychological overload, with possible implications for the perception of QOL and well-being at this stage of life^(^ 43 ^)^.

As for QOL, the elderly presented higher means in the WHOQOL-BREF “Social Relationships” domain and in the WHOQOL-OLD “Death and Dying” facet. These results were similar to those of studies conducted in elderly care services^(^ 5 ^,^ 36 ^)^. The elderly presented lower means in the WHOQOL-BREF “Physical” domain and in the WHOQOL-OLD “Sensory Abilities” facet, as in another national study^(^ 36 ^)^.

There was a positive, weak and statistically significant correlation between the overall RS score and WHOQOL-BREF, and a positive, moderate and statistically significant correlation for WHOQOL-OLD. In the national and international literature, there is a scarcity of studies that used the same instruments to assess resilience and QOL in elderly people with NCDs and with preserved functional and cognitive aspects. A study on 120 American elderlies also assessed resilience using RS, but they used the Medical Outcomes Study 36 - Item Short to measure QOL. The results showed a weak and positive correlation between resilience and the domain of physical (r=0.24; p=<0.01) and mental health (r=0.38; p=<0.01)^(^ 44 ^)^.

In this regard^(^ 2 ^)^, the perspective of active aging, which involves the physical, social and psychological aspects that have an impact on the way one experiences old age, is also linked to QOL, and, among these aspects, resilience is an important dimension to be rescued or maintained, since it is a development tool of reserves needed to adapt to vulnerable situations in order to maintain life satisfaction during old age Therefore, investing in self characteristics, added to life-long experiences, strengthens the remaining regulatory mechanisms in the elderly.

From this perspective, a review study pointed out that the high degree of resilience among the elderly is related to the development of mental (adaptation, coping, gratitude, positive emotions, hope, among others), social (involvement with community, family and friends, social support, among others), and physical (mobility, independence, functionality, among others) characteristics^(^ 1 ^)^.

With regard to symptoms of depression, the physical, social and psychological demands related to aging may compromise the mental health of the elderly. Most of the elderly in this study (64.2%) presented symptoms of depression, assessed through CES-D. In general, the elderly receiving outpatient care have chronic diseases, relative degree of dependence and symptoms of depression^(^ 31 ^)^.

In this study, the relationship between symptoms of depression and resilience was negative and significant, and the mean score on RS was higher for the elderly without symptoms of depression. In Brazil, a study conducted with 59 elderlies receiving outpatient care, in the city of Campinas-SP, was aimed at describing the relationships between abilities, symptoms of depression and cognition in resilient and non-resilient elderly, using RS and the Geriatric Depression Scale (GDS). Results revealed a negative and moderate correlation (r = -0.688; p = 0.01) between resilience and symptoms of depression; as for the comparison between the means, the elderly with low resilience (≤ 66 median points) had higher GDS score (6.4; SD = 4.2) when compared to those with high resilience (2.6; SD = 2.6), with statistical significance (p = 0.001)^(^ 31 ^)^.

With regard to symptoms of depressions, it is known that stressful life situations and events, risk behaviors, behavioral excesses and negative emotions experienced throughout life are significant for the onset of these symptoms during old age^(^ 45 ^)^.

Thus, the maintenance of emotional balance regulation acts as a psychological attribute for those elderlies who can age actively, minimizing senile influences through resilient characteristics and self-efficacy developed throughout their lives^(^ 46 ^)^.

Given the above, less resilient elderlies may be more subject to symptoms of depression and poor perception of QOL in old age. In addition, low schooling and income as well as having a partner were relevant variables for less developed resilience in the sample studied.

Considering their theories and models of professional practice, nurses need to plan their assistance to address psychosocial aspects, recognizing risk and protection factors, with a view to developing resilient characteristics^(^ 47 ^)^. Therefore, from the initial assessment and establishment of the bond with the elderly, nurses should understand and include in their care plan the factors that will collaborate to promote these characteristics, and which enable better adaptation conditions of the elderly in the face of aging and occurrence of NCDs.

It is believed that the results presented in this study show the relevance of knowing the relationship between resilience, sociodemographic and health characteristics, QOL and symptoms of depression in the elderly receiving outpatient care. Thus, it is possible to provide necessary subsidies for the planning of care for the elderly, focusing on the behavioral mechanisms of coping, adaptation and overcoming, as well as with the purpose of stimulating and strengthening the development of positive attitudes along the aging process, favoring promotion of health and QOL.

The limitations of the study refer to the data only of the elderly attended at the outpatient clinic, that is, the characteristics are peculiar to the sample studied and generalizations should be considered with caution in order to avoid misunderstandings. No sample calculation was performed; therefore, the sample is not representative. In addition, the cross-sectional design did not allow long-term follow-up to assess resilience, QOL and symptoms of depression in the elderly.

## Conclusion

The results of this study showed that the elderly seem to be adapting and using resources and behavioral coping, adaptation and overcoming mechanisms, and that the variables schooling, income, symptoms of depression and QOL were related to resilience.

The emotional state of the elderly can contribute to the development of better resilient characteristics, which possibly will result in better physical and mental health, well-being promotion, pleasure, safety, and better understanding and acceptance of changes during aging.

In this sense and with regard to research in the field of nursing, the results of this study offer elements that may result in information that help in the elaboration and improvement of its practice, specifically in the elderly population, in order to strengthen coping, adaptation and overcoming in situations of disabilities and limitations that may arise with advancing age.
